# Assessment in ‘survival mode’: student and faculty perceptions of online assessment practices in HE during Covid-19 pandemic

**DOI:** 10.1007/s40979-021-00083-9

**Published:** 2021-08-10

**Authors:** Zilal Meccawy, Maram Meccawy, Aisha Alsobhi

**Affiliations:** 1 English Language Institute, King Abdulaziz University, P.O. Box 80200, Jeddah, 21589 Saudi Arabia; 2 Department of Information Systems, Faculty of Computer Science and Information Technology, King Abdulaziz University, P.O. box 80200, Jeddah, 21589 Saudi Arabia

**Keywords:** Online assessment, Covid-19 pandemic, Higher education, Cheating, E-learning, Blackboard®, Academic integrity

## Abstract

This paper presents a cross-sectional study that demonstrates how King Abdulaziz University has responded to the lockdown imposed by the Ministry of Education in Saudi Arabia due to the Covid-19 pandemic. The purpose of this study was to examine the perceptions of students and faculty towards assessment that had to take place online due to physical or social distancing rules and lockdowns. A descriptive mixed-method study was conducted with two different self-administered questionnaires that were developed for students and faculty, respectively. A total of 547 responses were received from undergraduate students and 213 from faculty. The main finding suggests the need for a multilevel approach to the problems of cheating and plagiarism, including raising student awareness and ethics, training teachers to detect cheating methods, and institutions activating their code of practice and applying severe sanctions on those who engage in such practices.

## Introduction & study background

The year 2020 started with a new virus that crossed the lines between human and animal and caused a worldwide pandemic. In a matter of months, everything was affected, and the world as we knew it changed. Physical distancing rules, government-mandated masks, and shut-downs, as well as partial and total lockdowns, were taking place in almost every country. The health sector was affected, as well as trade, the economy, the environment, and, certainly, education. As of March 9th, Saudi Arabia’s Ministry of Education (MoE) closed schools, universities, and colleges and moved learning completely online as Al-Samiri (2021, p.148) explains: “In a brief timeframe, the whole country began the transition to remote learning environments, whether it was televised on select channels or communicated through various online platforms: Telegram, Zoom, Teams, WebEx, and Blackboard”. King Abdulaziz University (KAU), specifically, moved learning from traditional classrooms on its various campuses to Blackboard®—an online Learning Management System (LMS) with both synchronous and asynchronous features. The university had to deliver its educational services to faculty and students in the spring term during this emergency crisis. Teachers and students alike had no choice in this matter, and many changes, including some to assessment practices, were necessary to survive the pandemic period without halting the educational process completely. In digital video games, this scenario parallels what is known as the ‘survival mode.’ In such games, the player must continue playing without dying in an uninterrupted session for as long as possible (the remainder of the semester) while the game (the learning environment, government regulations, and resources) provides players with increasingly difficult waves of challenges.

Despite the situation being far from ideal, “Saudi universities were naturally better prepared to transition to the online learning environment, as most Saudi universities had already implemented digital communication and learning tools” (Al-Samiri 2021, p.149). For several years prior to the pandemic, the university’s LMS, namely Blackboard®, was used for paid or executive online courses with external or distance learning students in some faculties, so faculty and staff had an awareness of it, and some might have used it, but not all had the same experience or level of knowledge of it and its features. “This software was not used extensively and served a supplementary role prior to the pandemic and its e-learning users are still discovering its features” (Al-Samiri 2021, p.149). For example, before the pandemic, the English Language Institute (ELI) at the university moved weekly assignments for Preparatory Programme Year (PYP) students online to Blackboard® and employed plagiarism detection software to writing assignments, but no tests or quizzes were taken on the system.

Online teaching started taking place via synchronous virtual classes using the university’s LMSs, mainly Blackboard® and Blackboard Ultra®, in the middle of the spring semester at KAU, which started on January 19th and ended on May 14th, 2020. However, for teachers and students in the Preparatory Year Program (PYP), the move to online teaching and learning was at the beginning of their fourth and final quarter. This study was designed after the MoE-mandated closures started, and data were collected between April and May 2020 towards the end of the second semester. As a descriptive mixed-method study, it was conducted on both male and female campuses at KAU, where undergraduate students of all levels, as well as academic staff with different rankings, participated by filling out online questionnaires for students and faculty, respectively. The questionnaires were designed to look at the online learning and teaching experience from three different angles or axes, namely (1) teaching and learning, (2) assessment, and (3) social and technical aspects. However, this paper will only focus on results from the second perspective, namely the assessment axis. The study is dedicated to exploring and answering the following questions:

1. How did the university handle assessment online?
2. What did students think of online assessment?
3. What did faculty think of online assessment practices?

### Theoretical and conceptual framework

As a result of the COVID-19 pandemic, learning has migrated online in most classrooms, colleges, and universities around the world. This has removed the alternative of combining informal learning with formal education as a choice from both students and teachers. Online learning and networking became the new standard, replacing conventional classroom teaching, and online testing has also been shifted. This happened with little preparation due to the new World Health Organization (WHO)-recommended physical distance rules that refused any group presence in a closed physical space, such as a classroom where the novel virus could spread. Hence, it was important “to make a transformational shift in [our] approach to teaching from one of disseminating information to one of creating learning environments where students [could] co-construct knowledge through interactions” (Vaughan 2010). This idea of co-constructing knowledge through student interaction informs the theoretical framework for this study, which is based on the premise that teachers need to encourage online learning communities among their students and allow them to make the connections between the different complex knowledge sets they encounter during their learning. Other learning theories do not do that as they “do not address learning that occurs out of people (i.e., learning that is stored and manipulated by technology)” unlike connectivism which states that “learning may reside in non-human appliances” (Siemens 2005).

### Literature review

Research has shown that teachers are engaged in assessment-related tasks for as much as one-third to half of their time (Stiggins 1992). Practitioners define educational assessment as the process of gathering information about student learning and has several types, methodologies, and approaches. Nicol (2008) argues that “[a]ssessment is said to drive student learning: it can provide the motivation for learning (e.g., through the awarding of marks and grades), but it also enables learning to take place through the provision of feedback.” It can take the shape of formative assessment, which takes place during a course to aid student learning, inform teachers of their teaching practices, and provide feedback. Its aim is to promote learning. As Sardareh and Mohd Saad (2013, p.2493) put it, “[r]esearch suggests that formative assessment can improve students’ learning. However, the concept of formative assessment does not still represent a well-defined set of practices and this issue might affect its successful implementation in different contexts.” Educational assessment can also take the form of summative assessment, which takes place at the end of a learning course to measure student achievement. Its aim is to measure learning. Summative assessment “is a high stakes assessment with a final mark of achievement awarded describing the learning achieved against public criteria” (O’Shaughnessy and Joyce 2015, p.201). In other words, “[i]t is a powerful tool in the armory of the educator and therefore, deserves careful consideration. When exploring assessment, there are six key questions which should be addressed; why, what, how, when, where and who” (Harden and Laidlaw 2012). This literature review will cover three aspects: (1) online assessment, (2) online cheating opportunities, methods, and reasons; and finally, (3) teachers’ perspectives of online assessment.

#### Online assessment

Moving assessment from a physical classroom environment to an online one is challenging because “often the temptation or commonly used approach is to mirror face-to-face strategies and practices” (Bailey et al. 2015, p.112). However, technology can offer assessment much more in terms of access and support in its various stages, namely task design, assessment or interpretation, and feedback and grading, as Nicol (2008) explains:

Virtual Learning Environments (e.g. Blackboard, Moodle) can make it easier to present assessment tasks to students (e.g. to publish task requirements, the criteria to be used in assessment and the timings for submissions) and to track and record student progress (e.g. automatic time logging of activities and assignment submissions).

Bailey et al. (2015) carried out a study of 35 US university students through an online survey seeking their perceptions of 12 assessment strategies included in an online course. Then, they analyzed the data in terms of engagement, enjoyment, and knowledge, and an overwhelmingly positive experience was indicated: “The overarching implication for this study is that online professors and online instructional designers must move away from their comfort zones in pursuit of more innovative instructional strategies and assessments that will engage students,. ..” (ibid., p.123). Conversely, a qualitative study by Khan and Khan (2019) of 41 university science students in the United Arab Emirates exploring student perspectives on online assessment revealed that students resisted online high-stakes assessments and had apprehension towards them due to several factors, such as personal preferences, technological competency, layout, cheating and subject discipline, and finally, grading and feedback. The study revealed that students in the study appeared not to understand “the usefulness of the transition to online assessments.. . [however,] students saw some advantages to online assessments, in the form of colored diagrams, being able to edit answers, spell check. ..” (Khan and Khan 2019, p.673).

#### Online cheating: reasons, methods, and possible solutions

A major problem of assessment present in traditional classrooms is cheating, or academic dishonesty. It is a long-standing problem around the world; for example, in the US, “[t]he first comprehensive study of cheating at colleges and universities (5,000 students at almost 100 institutions) was completed in 1964. It found that 75 percent of the students had engaged in one form or another of academic dishonesty” (Chace 2012, p.12). King, Guyette, and Piotrowski (2009, p.4) define cheating as “a transgression against academic integrity which entails taking an unfair advantage that results in a misinterpretation of a student’s ability and grasp of knowledge.” Cheating is a form of academic dishonesty and can vary in severity and method. A Canadian study investigating 412 faculty members’ attitudes towards student violations of academic integrity revealed that just over half of the respondents felt it was getting worse and that their institutions’ unenforced policies are one reason behind this (MacLeod and Eaton 2020).

In their research, Baijnath and Singh (2019) looked at several studies from over 14 countries examining research on cheating practices in Higher Education (HE), perceptions, and possible solution to the phenomenon, and they recognized cheating as an international problem, with technology as one of the main enablers of cheating, and the fundamental role universities play in combating this issue on a societal level. Academic dishonesty is present in traditional classrooms, but when assessment is moved online, the problem becomes more complicated. In their study, King, Guyette, and Piotrowski (2009, p.7) found that “[c]learly, the majority of the students held the belief that more cheating occurs in online courses that it is easier to cheat in an online versus a traditional course.” Online academic integrity is a major concern that universities must address due to “the increased potential of cheating since the instructors have no control over the test setting, thus are not able to monitor students taking tests” (Palloff & Pratt, cited in Kayed 2013, p.20). Therefore, serious considerations need to be given to assessment before hosting an online course because there are real issues of concern, such as the type of assessment, academic integrity, and test security. Quizzes and tests have always been used in traditional classrooms but are inappropriate and insufficient in online environments, as “they do not reflect the true capabilities of online students” Kayed (2013, p.20). He further argues that “there is no assurance that the enrolled student is actually the one who is completing the work. Moreover, there is always the possibility that students intentionally or intentionally will plagiarise by not giving credit for others’ work words and/or ideas” (ibid.). Finally, Heberling (2002, p.1), when referring to online education, states that “[a] major reservation seems to center on the issue of cheating and plagiarism in the online classroom.”

McCabe and Treviño (1993, cited in McCabe et al. 2001) reviewed a decade of cheating in academia and found that a school lacking an honor code had lower levels of cheating behavior, while a school with an honor code had a higher level. To explain this, they took a closer look at the schools, where they realized that the school lacking in a formal honor code “had developed a culture that emphasized many of the elements found at code schools and encouraged academic integrity without instituting a formal code” (McCabe et al. 2001, p.224).

Moreover, Larkin et al. (2017) conducted a study on 30 US university graduate students to investigate whether it was easier to cheat online, how they perceived plagiarism, and what were their impressions of university policy regarding academic integrity. They found out that most students believed that cheating takes place during testing, that it is easier to cheat online, and that they had a clear idea of what constitutes plagiarism, although some believed that copying from the internet was acceptable. It also showed that some students do not feel “cut and paste” is a problem, and, thus, “it is imperative that instructors explicitly address what constitutes unacceptable plagiarism-related behaviors” (Larkin et al. 2017, p.6). King, Guyette, and Piotrowski (2009, p.2) state that “[p]erhaps the online environment or milieu contributes to the temptation to use dishonesty (in its many forms) due largely to the lack of oversight on the part of instructors.” Hence, implementing an honor code, for example, should be part of the teacher’s responsibility.

Just as reasons for cheating vary, so do the methods employed: “Today, lots of students cheat. They use the work of others, they buy essays. They plagiarize. Still, even though the Web makes cheating easier than ever before, and thus more prevalent, the phenomenon of cheating is nothing new. Students have been at it for a long time” (Chace 2012, p.23). According to Olt (2002), methods for cheating “can be divided into two: those that require an accomplice and those that do not.” Larkin et al. (2017), p.2) state that “[cheating] includes collaborating on homework, using ‘cheat sheets’ during an exam, and plagiarizing assignments. In more extreme forms, academic dishonesty involves students purchasing term papers from paper mills.” Cizek (1999) mentions several ways of cheating, including looking at another student’s exam paper, exposing a test paper for others to cheat from, passing an eraser with answers written on it between students, developing codes, such as tapping the floor to indicate a specific answer, using small papers to cheat, writing test information on the desk, and using banned resources in take-home exams—to name a few.

Clark and Lancaster (2006, cited in Eaton and Dressler 2019) constructed the term ‘contract cheating’ which is when a third party is involved and “include[s] essay mills, homework completion services and professional exam takers (impersonators) among others. All forms of contract cheating are considered academic dishonesty, negatively impacting student learning and assessment” (Eaton and Dressler 2019, p.4). Contract cheating “falls squarely within the category of intentional academic misconduct” (Ellis, Zucker and Randall, 2018, cited in Eaton and Dressler 2019, p.4 .) and is typically carried out (online and offline) without the knowledge or approval of the instructor by third parties for reasons such as money, loyalty, and friendship (ibid.). However, as argued by Olt (2002), “[d]istance. .. does not diminish the possibility of students cheating, with or without an accomplice, on online assessments.” According to Heberling (2002), “[t]he Internet made it very easy to cheat in any classroom setting (traditional or online). Students can cut and paste lengthy passages from multiple Internet sources and then splice them together for term papers. This cut and paste technology makes cheating so easy that the students get both lazy and sloppy.”

Finally, another method of cheating mentioned by Heberling (2002) is using ‘digital paper mills,’ where students can buy written papers, sometimes even for free, through websites advertising revenue. He argues that these mills are a problem for both traditional and online classrooms; however, if submitted online, they are easier to detect than if they were handed in by hand in the classroom.

The internet and technology might have increased the temptation to cheat; however, on the positive side, there are tools to help combat online plagiarism, which “allow the instructor to search and compare large portions of a student’s paper with material available on the Internet” (Heberling 2002). For example, on Blackboard®—the LMS used by KAU during the lockdown—if the instructor enables the SafeAssign tool, then any submission by the student would be analyzed against the institutional database and the internet, which makes it easier to detect plagiarism by producing an originality report providing the original sources of students’ work. It supports several languages, including Arabic and “uses algorithms that make decisions about the originality of the submitted text. The algorithms consider word frequency, sentence structure, and other linguistic characteristics” (Language Support | Blackboard Help 2021). Heberling (2002) argues that, “[a]s a deterrent, it might be worthwhile to let students know that the Internet can (and will) be used to combat plagiarism. .. [and that it] is unacceptable[,] and severe consequences up to and including expulsion await those students who wish to test the policy.” He also adds that the administration should stand by faculty who impose these ethical standards of academic integrity and work together toward minimizing cheating and plagiarism (Heberling 2002) in both traditional and online learning.

In addition, to overcome these issues of concern, Kayed (2013) suggests that online teachers should devise further safeguards to ensure integrity, such as the use of multiple assessment techniques, designing online take-home exams where students’ knowledge rather information recollection is tested, using various software to detect plagiarism, and arranging controlled exams sittings at the university or a common location. However, this final solution is not feasible nor recommended in times of crises such as the COVID-19 pandemic and its subsequent lockdowns and government-mandated rules for social distancing.

#### Online assessment: teachers’ perspectives

Educational assessment is usually divided into two approaches: summative and formative, where summative assessment measures student achievement at the end of a course, or a semester, usually for the purpose of writing a report or awarding a grade (Black and Wiliam 1998) and formative assessment is perceived as a diagnostic tool to provide feedback during the learning process and is “aligned with constructivist-based teaching approach which involves active learning activities such as open-ended problems, observations, interviews, writing samples, exhibitions, and portfolios,” as argued by Sulaiman et al. (2019, p.426). In their study of teachers’ perceptions of assessment and alternative assessment in the classroom, Sulaiman et al. (2019, p.430) concluded that “[t]o assess students’ knowledge and skills, teachers need to implement several assessment instruments such as writing test, project, assignment, simulation, portfolio, journal, exhibition, observation, interview, oral exam, and peers evaluation.” Alvarez et al. (2009, p.322) argue that “teaching and learning in virtual environments imply making changes to the organization of teaching and, subsequently, a change in the teacher functions” and that “[o]nline teaching and learning requirements are not limited only to a set of knowledge and experience; the challenges a teacher faces are linked closely to the particularities of interacting and communicating online”. These teacher roles expand because “[i]nstructors can no longer depend on different handwriting, a change in ink color, or the detection of eraser marks on an assessment as evidence that a student has changed answers after having taken the assessment” Olt (2002) and they need more training to be able to carry out assessment successfully and detect cheating online in other ways. On the contrary, data collected by Mellar et al. (2018) in an exploratory study at two universities, one in Turkey and one in Bulgaria, from three groups of participants (administrators, teachers, and students) to find common views and differences between traditional and online contexts revealed that teachers’ opinions across all contexts were comparable, and differences were due to reluctance to depart from an established, secure, and large-scale assessment system.

In her study conducted on 53 prospective and 47 practicing English language teachers to explore how teachers conceive language assessment using metaphors and whether these conceptions differ according to teaching experience, Sahinkarakas (2012, p.1787) argues that “[f]or assessment to have an impact on student achievement, teachers need to see [it] as an integral part of the instructional process rather than as an evaluation device to determine students’ grades.” Her findings show that although high-stakes tests are used, teachers perceive assessment as a formative tool to enhance learning and value its contribution to improving instruction. Furthermore, teaching experience plays a minor role in their conceptions. Despite her study not being about online assessment, it clearly shows the importance of formative assessment, which was what the MoE focused on regarding grades during the pandemic.

A study by McCabe (1993, cited in McCabe et al. 2001, p.225) reinforced some student views that many faculty members do not take cases of academic dishonesty seriously: “For example, more than half of the noncode faculty reported that their most likely reaction to an incident of cheating would be failure on the test or assignment involved (39%), a simple warning (9%), various penalties less than test or assignment failure (7%), or nothing (1%).” Hence, faculty members tend to deal with cheating instances themselves without institutional interference, either because they do not wish to deal with the issue or fear a lack of support from their institutions, which becomes harder in an online environment. As McLeod and Eaton (2020, p.357) explain, “[t]herein lies the paradox of faculty attitudes towards dealing with academic dishonesty: most faculty members report that it is one of their key responsibilities, yet they often avoid confronting it.” Chace (2012) explains:

Some teachers know when a student's work is fraudulent but elect to do nothing. It takes time, and time is expensive; bringing a student before a campus judicial council is also labor intensive, and the outcome is unpredictable; students or their parents can retain attorneys to fight the charges and endlessly complicate the procedure; administrators cannot be counted on to back up professors making accusations. Professors like the elevation of teaching but not the grubby business of prosecuting.

## Methods, design and data collection

To collect data to answer this study’s research questions, two online questionnaires were designed, created using Google Forms, and distributed to students and faculty via WhatsApp, email, and Blackboard®. Undergraduate KAU students were randomly contacted through their instructors via university emails, LMS communication channels, and student-teacher WhatsApp groups, as well as the university’s MyKAUApp. A similar sampling procedure was applied to faculty, who were randomly selected utilizing official university emails and shared staff mobile chat applications. All responses were anonymous and confidential; however, faculty were given the option to provide their email addresses, and just under 40% (*n* = 83) provided their university emails. At the beginning of both questionnaires, a short paragraph explaining the study’s aims and objectives was provided to assure potential participants of anonymity or confidentiality. This was followed by a question of consent, where participants were asked to confirm that they were either students or academic staff at KAU and that they agreed to participate in this study, and answering “No” to either option resulted in the termination of the questionnaire and no data being collected. The sampling was purposeful; (*n* = 547) students participated in the research, as well as (*n* = 213) faculty members. Any postgraduate, diploma, paid or executive degree students were either not contacted or excluded and a small pilot was carried out with students to check understanding and with faculty to ensure translation was accurate.

### Students

The student questionnaire, which was in Arabic, contained 45 questions and was divided into three main sections preceded by an introductory section gathering student demographic data through four questions about their gender, discipline, year, and Grade Point Average (GPA), as shown in Table 1 below. The other three sections looked at different aspects of their online learning experience, which were: (1) social and technical aspects (13Qs), (2) teaching and learning aspects (13Qs), and finally, (3) those aspects concerning online assessment (14Qs). This was in addition to one last question regarding their overall online learning experience at KAU. This paper only provided results for data related to the online assessment section as well as the final overall question. A wide range of students responded to the questionnaire, all of whom were assumed to have had the same guidance on academic integrity and institutional policy during their orientation week at the start of their academic year (before the emergency shift to online learning) due to the pandemic lockdown since “[f]ew students are ignorant of the prevailing ethical standards of their home institutions.” (Chace, 2012, p.26).

**Table 1:** Student Demographic Data

Category	Frequency	Percentage
**Gender**		
Male	172	34.4%
Female	374	68.4%
**Discipline**		
English Language Institute (Preparatory Year)	188	34.4%
Faculty of Computing & IT	155	28.3%
Faculty of Engineering	45	8.2%
Faculty of Science	42	7.7%
Faculty of Medicine	42	7.7%
Faculty of Economics and Administration	23	4.2%
Faculty of Arts & Humanities	18	3.3%
Other Faculties	34	6.2%
**Year**		
Preparatory Year (1st or 2nd semester)	188	34.4%
Year 2 (3rd or 4th semester)	132	24.1%
Year 3 (5th or 6th semester)	79	14.4%
Year 4 (7th or 8th semester)	79	14.5%
Year 5 (9th or 10th semester)	61	11.2%
Year 6 (medicine only)	8	1.5%
**GPA**		
4.5 and above (A & A+)	184	33.6%
3.75–4.49 (B & B+)	244	44.6%
2.75–3.74 (C & C+)	94	17.2%
2.74 and lower (D & D+)	25	4.6%

### Faculty

A 5-point Likert-scale questionnaire was presented to faculty both in Arabic and English (for international staff/ non-Arabic speakers). The questionnaire included 47 questions and, like the student questionnaire, was divided into three main sections preceded by an introductory section gathering faculty demographic data, such as gender, discipline, ranking, teaching experience (in years), and email address (optional), as shown in Table 2 below. The other three sections looked at different aspects of their online teaching experience, which were: (1) social and technical aspects (15Qs), (2) teaching and learning aspects (13Qs), and finally, (3) those aspects concerning online assessment (13Qs). There was an open-ended option for any comments or suggestions for faculty to avail of if they so wished. This paper only provided results for data related to the online assessment section as well as any relevant qualitative data from the open-ended comment section.

**Table 2:** Faculty Demographic Data

Category	Frequency	Percentage
**Gender**		
Male	42	19.7%
Female	171	80.3%
**Discipline**		
English Language Institute	74	34.7%
Faculty of Computing & IT	55	30.5%
Faculty of Science	30	14.1%
Faculty of Dentistry	10	4.7%
Faculty of Arts & Humanities	9	4.2%
Faculty of Economics and Administration	8	3.8%
Faculty of Medicine	5	2.3%
Other Faculties	22	5.7%
**Ranking**		
Professor	16	7.5%
Associate Professor	25	11.7%
Assistant Professor	72	33.8%
Lecturer Professor	37	17.4%
Teaching Assistant	10	4.75%
Language Instructor	38	17.8%
IGIT	7	3.3%
Other (Temp)	8	3.75%
**Teaching Experience**		
One year or less	9	4.2%
2–5 years	48	22.5%
6–10 years	46	21.6%
10+ years	110	51.65%

## Data analysis and results

### Student quantitative data

The student questionnaire had 15 closed questions, five of which had an open-ended option (Qs: 4, 8, 13, 14, & 15). All 15 questions were related to student perceptions of assessment practices, which included: (1) change in assessment practices, (2) nature of this change, (3) effect of this change upon workload, skill-based subjects or applied sciences, GPA, and psychological well-being, (4) perception of this change, (5) cheating—opportunities, actual cheating, reasons, methods—and (6) overall online experience.

As demonstrated in Table 3 above, 64.8% of students stated that there was a change in assessment practices after the move online, and that the nature of this change was mainly in the division of grades (88.5%). Students felt this change was in their favor, but increased their workload (41.2%), was positive and will improve their GPA (45.5%), while also having a negative effect on their anxiety levels and general well-being (56.8%). When surveyed about applied sciences, 30.9% stated they did not have any such subjects, while those who did stated they assessment changed into more formative assessment methods (29.6%) and skill-based subjects were carried out synchronously via Blackboard® or Zoom (48.7%). Students’ perception of this change revealed that 33.8% felt assessment practices were strict with them, while 30.8% felt they were lenient and 45.2% felt less anxious due to being home in familiar surroundings. The solution to the change in circumstances was to continue with the same grade division of the MoE (31.3%). Questions regarding cheating online showed that 48.1% reported that there were not ample opportunities for cheating, and the majority (16.1%) of all who cheated on assignments (total of 34.5%) reported doing so in only one assessment. Of those who cheated (*n* = 217), the majority with 20.3% stated the difficulty of understanding lessons online as their reason, with ‘ease of cheating online’ (18.9%) and ‘general anxiety of the situation’ (18%) as other reasons. Using WhatsApp for cheating online was their number one method (27.4%) and the phone as their least chosen method (1.3%). Finally, 47% believed their overall online experience was successful despite the challenges, while 32.75% stated that the experience was equally positive and negative.

**Table 3:** Students’ Self-reported perceptions of online assessment practices during COVID-19

Question	Response (***n*** = 547)
**(1) There was a change in assessment practices in one or more subjects**	
Yes	64.8%
No	9.2%
Some	19.7%
Don’t know	6%
**(2) If there was a change in assessment practices, the nature of this change was**^**a**^	
Change in grade division only	88.5%
Change from paper-based to Computer Based Tests (CBTs)	60.8%
Change in the nature of assessment itself	42.7%
No noticeable change	3.6%
**(3) If there was a change in assessment practices, as a student, I felt that it was**	
In my favor and lightened the workload	18%
In my favor, but increased the workload	41.2%
Not in my favor, as workload increased & grades affected negatively	30.7%
Neutral/ no opinion	10%
**(4) If there were practical subjects (such as labs, studios, etc.) the assessment was**	
Fully theoretical via homework & quizzes	29.6
Practical via virtual classrooms/ online	28.5
Cancelled/ postponed	7.7
Had no practical subjects	30.9%
Other (open-ended)	3.3%
**(5) Subjects requiring direct interaction between student & teacher (such as speaking/ reading in language learning), the assessment was**	
Carried out synchronously/ Online via Blackboard®, Zoom, etc.	48.7%
Cancelled/ replaced	10.6%
I didn’t have any such subjects this term	40.8%
**(6) Regarding online assessment (synchronous and asynchronous), my opinion of it was**	
Better because I was home and felt comfortable (less stressed than exam hall)	45.2%
Worse because of distractions, unlike in an orderly exam hall	23.2%
Worse because of technical issues at home (devices and internet)	17.7%
Worse because of my lacking technical skills or knowledge	11%
No difference	11.9%
**(7) I felt that student assessment practices during the crisis, compared to pre-crisis, were**	
More lenient with students	30.8%
Stricter with students	33.8%
No difference	14.1%
Not sure/ I don’t now	16.5%
My first semester, so I cannot compare	4.8%
**(8) In my opinion, the best solution to assessment during the crisis was to**	
Use semester 1 grades	12.1%
Continue with the same grade division (of the MoE)	31.3%
Not count semester 2 in the GPA, just get pass or fail	23.2%
No opinion	23.2%
Other (open-ended)	10.2%
**(9) Personally, I found the changes to assessment practices during the crisis**	
Positive and will improve my GPA	45.5%
Negative and will lower my GPA	16%
No change to my GPA	13%
I’m not sure yet	25.6%
**(10) The new assessment practices (online) had a psychological effect on me as a student**	
Negative effect (anxiety/ fear of tests)	56.8%
Positive (no anxiety/ fear of tests)	25.6%
No noticeable effect	17.7%
**(11) There were ample opportunities for cheating on exams this semester**	
Yes	20.9%
No	48.1%
Not many	31%
**(12) When it came to cheating online, I**	
Cheated in all assessments	3.4%
Cheated in some assessments	15%
Cheated in one assessment only	16.1%
Didn’t cheat in any assessment	65.5%
**(13) If I cheated, it was because of (*n*** **= 217)**	
Ease of cheating online	18.9%
Difficulty of understanding lessons online	20.3%
No time to study	6.4%
Not in the mood to study	3.2%
Fear of failing subjects	10.6%
General anxiety of situation	18%
Other (open-ended)	12.9%
**(14) In my opinion, the most common method of cheating among students was**	
WhatsApp	27.4%
Internet	6%
Phone	1.6%
Identity impersonation	3.1%
I don’t know	57%
Other (open-ended)	4.9%
**(15) Finally: in general, the online learning experience at KAU during the covid19 crisis/ lockdown was in my opinion**	
Successful overall despite the challenges	47%
Unsuccessful despite some positives	11.9%
Equally positive and negative	32.7%
No opinion	4.7%
Other (open-ended)	3.7%

^**a**^More than one option could be chosen

### Student qualitative data

The qualitative data obtained through the open-ended options in some questions were analyzed using thematic analysis by applying the five steps identified by Braun and Clarke (2006), which are: familiarization with the data, assigning initial codes to describe the content, searching for themes, defining, reviewing and naming themes, and producing the report. These data, all of which were translated into English (then checked by a faculty in the English Language Institute (ELI) for validity) yielded the following themes: (1) change to assessment of skill-based subjects or applied sciences and nature of this change, (2) student perception of the best solution to assessment during the crisis, (3) reasons for cheating or not cheating, and (4) cheating methods used. In the final question regarding their overall online experience, the themes were: (a) positive experience and (b) negative experience. However, an emergent theme from student qualitative data was: (c) equally positive and negative, especially when related to the Preparatory Year Programme (PYP) and included issues concerning grades and tests being extremely high stakes. Given the high number of participants from this category (*n* = 188, 34.4%), it was deemed an important theme (see Table 4 below for detailed themes and subthemes).

**Table 4:** Themes from Student Qualitative Data

#	Statement/Question	Theme(s)	# of Mentions
**Q4**	Change in assessment practices in skill-based/applied or interactional subjects	No change/no such subjects	7
		Nature of the change	6
**Q8**	Best solution to assessment during the crisis	Grades/GPA	27
		Teacher-related (help/leniency)	7
		Exams	6
		MoE’s decisions	5
**Q12**	Reasons for cheating (or not cheating)	Religion (no cheating)	7
		Exam time limit	6
		Difficulty of cheating online	5
		Type/features of exam	4
		Combination of reasons	2
		Stress & anxiety	1
		Ease of tests	1
		Ability to cheat	1
		Teacher complacency	1
		Necessity/self-preservation	1
**Q14**	Most common method of cheating among students	Combination of methods	4
		Reference to textbook	3
		WhatsApp and internet	3
**Q15**	Overall online learning experience during the crisis	Negative (faculty/anxiety/cheating & inequality/grades/connection problems/no well-being considerations/unclarity & confusion/communication)	7
		Positive (could be developed into something permanent/no need for physical attendance/ease of attending lectures)	5
		Both positive & negative aspects (especially negative for Preparatory Year Program students)	4

When discussing assessment practices for skill-based subjects, applied sciences, or subjects that require teacher-student interaction, such as languages (Q4), students explained the nature of change that took place during the pandemic lockdown. Some of these changes involved the type of assessment or homework required. For example, some students said that they were asked to send videos or submit a commentary on a YouTube video or simply send the work electronically. One student explained: “We were asked to shoot a video from home while working on a project,” while another stated that “[practical assessment] was replaced by theoretical assignments and discussions.” However, some students clarified that there were no changes to their skill-based subjects’ assessment, as “all laboratory work was completed before the lockdown,” and their “grades up until the lockdown were considered enough, and [they] had no more assessments,” or they did not have such subjects as part of their learning.

When asked about their perception of the best assessment solution during the pandemic (Q8), Four distinct themes emerged: faculty-related issues, MoE’s decisions, exam-related issues, and grade-related issues, with grades being their biggest area of concern. Students seemed to expect faculty to “help,” “assist,” “cooperate,” and make things “easy” for them due to the lockdown and the uncertain times they were living in. They believed that the faculty should “help students and alleviate pressure for [them],” “help and make it easier for students and this did not happen,” and “be lenient with marks to raise student GPA.” Students also wanted faculty “to increase homework while easing and facilitating exams.” In addition to suggesting that faculty helped them with assessment issues as a solution to the changes taking place, the majority believed the MoE’s decisions regarding grades were the best solution and were acceptable to them. The MoE decided that 80 marks were awarded to assignments, homework, and projects and 20 for the final exams, thereby adopting a more formative assessment approach as a solution to the unplanned complete move online during the crisis. Students agreed that that was the best approach to handle the change. One student commented that the best solution was “[t]o follow the decision of the Minister of Education. I felt the most appropriate thing was what the university did,” while another stated that “[t]he issue was resolved by the Ministry of Education,” and another claimed that “[t]he Ministry’s system is very fair.” The rejection of the proposed adoption of the first semester grades at the beginning by the university was reiterated, as one student explained: “The best way is the current way—online assessment. I believe that the adoption of first semester grades is a great injustice; please do not do that. This semester will increase my GPA, God willing.” Canceling final exams was the most commonly proposed solution to the assessment issue. Specifically, some students believed that they should get the final 20 marks by submitting a small project or homework online, whereas the majority believed that they deserved the remaining 20 marks and should be awarded to them without submitting any work or taking a final exam due to the circumstances. The best solutions, in their opinion, were “[t]o replace final exams with short tests or assignments,” “[t]o cancel the final and adopt 80 for pass and 40 for fail,” and “[t]o cancel the final exam and grant students the remaining 20 marks.” However, one student remarked that the best solution was “[t]o postpone the semester or transfer the exams to after the end of the crisis because the grades [were] not fair, and the reason is students’ cheating.”

However, students’ biggest grievance was their grades and GPAs (see Table 4 above). They mentioned the words “grades” and “marks” 27 times (the highest by far of all mentions in the student qualitative data). Some suggested being awarded bonus marks due to the novelty of the learning environment: “[the solution is to] award students a specific bonus mark for each subject because of the gaps caused by the confusion and strange situation of moving to online learning and assessment, which affected students compared to the beginning of the semester, so the bonus mark replaces or fills this gap.” Other students offered more unrealistic and impractical solutions, especially considering the massive number of students on all the university’s campuses: “[The solution is to] give students the freedom to choose; do they want to pass or fail or to calculate the GPA? If they achieved grades that raise the GPA, they would benefit, and if not, it does not hurt.”

Surprisingly, some responses show that some students were either unaware of the MoE’s decision that was in their favor or did not understand how it affected them, as demonstrated by the following quotes: “[I suggest that,] [i]n the event that the students’ grade in this semester was higher, it is calculated and vice versa, as adopting the first semester grades is considered an injustice to students who could not cope with the exams of the preparatory year, especially since it was somewhat difficult, especially physics,” which is exactly what the MoE decided to do.

When citing reasons for cheating online (Q13), students provided 10 explanations, including religion (for not cheating), exam time limit, difficulty of cheating online, type/features of exam, combination of reasons, stress and anxiety, ease of tests, ability to cheat, teacher complacency, and necessity/self-preservation.

When asked about the most common cheating method, (Q14), they mentioned using “a combination of methods,” “reference to textbook,” and “WhatsApp and the internet.”

In the final question (Q15) about overall online learning experience during the crisis, students either perceived it as a negative experience citing several reasons (faculty, anxiety, cheating & inequality, worry about grades, technological and connection problems, no considerations for their well-being, unclarity & confusion, and finally poor communication with faculty and classmates) or viewed it as a positive experience that could be developed into something permanent because there was no need for physical attendance, which created an ease of attending lectures, while a few saw it as an experience with equally positive and negative aspects (especially negative for PYP students due to the extremely high-stakes nature of their exams in terms of grades outcome and life-altering decisions that would be based on their scores).

### Faculty quantitative data

The faculty Likert-scale questionnaire had 13 questions related to online assessment that revolved around the following three major areas or themes, as shown in Table 5 below. Data were combined into three nominal categories: agree, neutral, and disagree.

**Table 5:** Faculty Quantitative Data

#	Theme	Sub-theme		Agree	Neutral	Disagree
**1**	Experience with online assessment	Pre-Covid-19: Types of online assessment	Formative	42.7%	4.1%	53.1%
			Summative	17.8%	4.7%	77.4%
		During Covid-19:	First time	51.7%	1.9%	46.5%
**2**	Challenges of online assessment	Constant change of grade division added to workload		76.5%	12.7%	10.9%
		Student GPA stability affected classroom interaction positively		53.7%	28.2%	28.2%
		Student Denied Final Grade (DN) awareness affected attendance negatively		67.2%	20.2%	40.9%
		Cheating was due to lack of proper invigilation		87.8%	10.3%	1.9%
		Grade inflation was due to cheating		77.4%	15.5%	7%
		Grades did not reflect student performance		59.6%	21.6%	12.2%
**3**	Traditional vs Online assessment	Convenience of online assessment auto-correct features		84%	9.4%	1.4%
		Unsuitability of online assessment of practical or skill-based subjects		61.5%	18.8%	15.9%
		Online assessment differed in quality & method		78.9%	13.6%	7.5%
		Preference for traditional assessment		46.4%	29.6%	23.9%

As seen in Table 5 above, just over half of faculty (51.7%) admitted that during the move to online learning due to the Covid-19 pandemic, it was their first time using Blackboard® LMS for assessment in general, while 42.7% said they had used it before Covid-19 for formative assessment only. However, the overwhelming majority (77.4%) had never used it for summative assessment. Among the many challenges faced by faculty, cheating was by far the most severe, where 87.8% felt that it was due to lack of proper invigilation, caused grade inflation (77.4%), and did not reflect student performance (59.6%). They also agreed that the constant change in grade division added to their workload (76.5%), they felt that student awareness of the stability of their GPA affected their interaction positively (53.7%), while 67.2% believed that student awareness of the impossibility of receiving a DN (denied final grade due to exceeding allowed number of absences) affected their attendance negatively. When comparing traditional and online assessment, 84% enjoyed the convenience of online assessments auto-correction feature, while 61.5% felt online assessment was not suited to practical or sill-based subjects. They also believed that online and traditional assessment differed in quality and method (78.9%) with 46.4% preferring traditional assessment methods.

### Faculty qualitative data

At the end of the questionnaire, there was a final open-ended question for further suggestions to improve teaching and learning during lockdown on any of the three aspects of their online experience: social and technical, teaching and learning, and finally those aspects concerning online assessment. Faculty provided (*n* = 78) responses in that section; (*n* = 14) of them revolved around assessment (18%). Faculty qualitative data surrounding assessment yielded three major themes: perceptions of assessment, cheating (security measures, technology, plagiarism detection), and teacher power, flexibility, and knowledge (training and workshops, grade changes/divisions). Their comments and suggestions were translated from Arabic where necessary (see Table 6 below).

**Table 6:** Faculty Qualitative Data

#	Themes	Sub-themes	Quotes
1	Perceptions of Assessment	Challenges	• “Student assessment [online] is a big challenge.” • Cheating was the biggest problem I had (Arabic)^a^ • [I suggest] [a]dopting better and stronger evaluation methods in distance education (Arabic) • The only real difficulty that we encountered is in the evaluation and tests (Arabic)
2	Cheating	Security measures	• [There is a need to] verify the identity of the students (Arabic) • A way to avoid fraud [is needed] (Arabic) • [We need to] develop a method for evaluating students to prevent cases of cheating (Arabic)
		Technology	• “Everything was perfect in terms of teaching and curriculum delivery, but parts of the assessment were problematic not because they are problematic but because the university refuses to employ security measures such as using CAMs [cameras] that would make the experience totally different and I, of course, mean better, here.” • [I suggest] [p]utting more restrictions in the programs to prevent cheating by male and female students (Arabic)
		Plagiarism detection	• “I faced difficulty on how to control the amount of plagiarized works as well as grading them fairly. Many would believe that zeros are the cure, but I think that [there] must be other solutions for this ethical issue.”
3	Teacher power, flexibility, & knowledge	Training/ workshops	• “I would recommend workshops for teachers on how to increase homework’s quality and other requirements presented by students.”
		Grade changes	• “Testing of writing and speaking skills should be improved by making them more transparent.” • “[Assessment] should be more flexible in terms of duration and writing our own exams.” • Give powers to the teacher in evaluating students with what he or she deems appropriate and not limiting the methods of evaluation (Arabic) • [There is a need to] establish attendance and participation scores to encourage further interaction (Arabic)

^a^Translated from Arabic

In the qualitative data (see Table 6 above), assessment was perceived by faculty as a challenge and cheating its main problem. When commenting on cheating, some faculty criticized the lack of security measures due to cultural or bureaucratic issues that prevented them from using cameras in assessment, especially to verify student identity. They also expressed the need for better plagiarism detection tools. In addition, some faculty expressed the need for more training on online assessment and cheating prevention measures as well as flexibility regarding grade changes. They wanted the power to award grades for attendance and virtual classroom participation, which they did not have as instructors, especially in the Preparatory Year Program -- an ongoing issue prior to COVID-19 due to standardization and concerns over test security (see Mansory and Meccawy 2017).

However, preventing cheating and plagiarism was a primary concern for faculty. There were several comments (*n* = 6) regarding finding ways to deal with cheating on exams and assessments, which, according to many teachers, had increased during this period. This could be achieved, in their opinion, by giving instructors more flexibility concerning assessment methods. One quote reads, “I faced difficulty on how to control the amount of plagiarized works as well as grading them fairly. Many would believe that zeros are the cure, but I think that [there] must be other solutions for this ethical issue.” Due to social and cultural restrictions, by default, webcams were not used neither by the staff nor by the students at the tertiary level, which made verifying student identity harder, as one faculty member wrote: “Everything was perfect in terms of teaching and curriculum delivery, but parts of the assessment were problematic not because they are problematic but because the university refuses to employ security measures, such as using CAMs [cameras] that will make the experience totally different, and I, of course, mean better, here.”

## Discussion

This study attempted to answer three questions related to online assessment during the COVID-19 pandemic lockdown: (1) How did the university handle assessment online? (2) What did students think of online assessment? and (3) What did faculty think of online assessment practices?

As of March 8th, 2020, the university moved all learning and teaching online, using the LMS Blackboard® at the start of the government-mandated lockdown due to the COVID-19 pandemic. Online assessment became a major debate point until the MoE decided that all testing was going to be online and courses were to follow a more formative approach to assessment, with most grades awarded as coursework, projects, and short quizzes. This was perceived differently by both faculty and students because it increased both their workloads but was in the best interest of student grades and marking. This is in line with the literature, where Heberling (2002) states that “[s]tudents who have taken courses in both traditional and online formats continually say that online courses require far more work,” which is what the student participants in this study expressed in the qualitative and quantitative data when asked about the workload increasing as a result of moving to a more formative approach to assessment. For example, students felt that it increased their workload, as they had many assignments to submit, but in comparison to high-stakes summative assessments, where cheating was possible and more common, it was fairer. McCabe, Treviño and Butterfield (2001, p.220) paint a bleak picture when they argue that

Students who might otherwise complete their work honestly observe this phenomenon and convince themselves they cannot afford to be disadvantaged by students who cheat and go unreported or unpunished. Although many find it distasteful, they too begin cheating to “level the playing field.”

One reason behind student cheating and academically dishonest practices could be due to internal pressure as well as external pressure as McCabe, Treviño and Butterfield (2001, p.220) argue that “[w]ith increasing competition for the most desired positions in the job market and for the few coveted places available at the nation’s leading business, law, and medical schools, today’s undergraduates experience considerable pressure to do well”. Interestingly, those who did not cheat did so for purely religious reasons since they felt cheating and academic dishonesty clashed with their beliefs even if they were disadvantaged by others who cheated.

Not only students’ workload increased due to emergency move to online teaching and learning, but faculty found themselves doing more. Alvarez et al. (2009) argue that in virtual teaching environments, teachers’ roles increase to include planning and design roles, social roles and instructive roles, which overlap. Furthermore, each of these roles has its own set of required competencies., which may explain why teachers felt that their workload remarkably increased after teaching moved online, as gleaned from the qualitative data. For example, instructors “have had to take on a technical support specialist’s role, teaching students, among other things, how to download, upload, and share their work” (Al-Samiri 2021, p.151). However, they were mostly concerned about academic integrity and how to maintain it by combating online dishonesty. This is because one of the main disadvantages of online assessment is the instructor’s inability to ascertain who is actually taking the test (Olt 2002), and anything that could affect the achieved score, such as cheating, will affect its meaning and overall validity. In a recent study by Reedy et al. (2021), they found that staff felt cheating online was easier for students and were concerned. Despite this prevailing notion that cheating online is easier, Heberling (2002) argues that it is easier to expose and even fight fraudulent work online since it is easier to detect it, whereas “[i]n a traditional class, the instructor does not have the benefit of seeing ongoing written products form each student,” and it is easy to compare student work in different assignments and student work across all sections. Since Blackboard® has an online plagiarism-detecting feature available for faculty, it might not be what they were referring to in the data when complaining about plagiarism; more likely, they were questioning how to deal with plagiarized work in the sense of how to score it or prevent it from happening in the first place, which is where they suggested the need for more training: “[d]espite the availability of anti-cheating software and plagiarism tools, not all instructors are adequately trained to apply them” (Al-Samiri 2021, p.152).

Finally, this study has several pedagogical implications, especially regarding assessment online and combatting cheating, which affects score meaning and, therefore, validity. Eaton and Dressler (2019) argue that one way to combat contract cheating is for teachers not to reuse assignments and to design in-class performance assessment tasks where students can demonstrate their skills, abilities, and knowledge as well as building their awareness of such methods. Furthermore, they believe that training is important, where “[e]ducator professional development is an essential element of building teachers’ understanding of contract cheating” (ibid., p.13), which happens in both traditional and online classrooms. McCabe, Treviño, and Butterfield (2001, p.231) argue that a clear message should be sent to both faculty and students “that cheating will result in negative consequences, and more than just a slap on the wrist” and support given to faculty who report instances of dishonesty. Furthermore, Chace (2012, p.26) agrees that

Few students are ignorant of the prevailing ethical standards of their home institutions. Should those standards be strong and consistently enforced, and should those institutions provide example after example of moral courage, students who cheat do so with the knowledge that they are violating a code of honor that has substance.

Olt (2002) suggests a few changes to assessment types and online approaches to tackle this issue, including creating numerous, short assessments throughout the course, making it difficult for students to solicit help throughout an entire course, creating open-book assessments of a substantive nature, and requiring student work to be submitted electronically, so that instructors can employ plagiarism tools. She also suggests setting time limits and attempt limits and randomizing online questions(Olt 2002; Reedy et al. 2021), which students in this study commented on as being a deterrent from cheating. Student data reveal that all of these methods seem to have been employed by different faculty. However, due to the emergency move to online teaching, these strategies might not have been properly planned and implemented at those uncertain times.

To overcome the problems associated with online assessment, such as cheating and plagiarism, we need to adopt a multilevel approach. Therefore, building on the results of the present study, we recommend applying such an approach to include the cultural and institutional context as well as policy regarding academic dishonesty. This is echoed by Olt (2002), who offers a three-tiered approach as a solution to the problem: (1) develop students that do not want to cheat (virtues approach), (2) eliminate and reduce opportunities or pressure for students to cheat (prevention approach), and (3) catch and punish those who cheat (police approach).

First, students need to be made aware of the severity of this practice on a moral and ethical level, and faculty could emphasize moral and/ religious teachings reflected in the data as a deterrent from cheating even when students have the opportunity. As Chace (2012, p.31) argues that “[e]very student on these campuses is informed, directly and formally, what honor means and why is it important” and “[t]hey see the dangers of cheating for what they are: practices in which many students can be hurt by the dishonesty of a few. And not just students but,.. ., the university as a whole, and the larger society beyond the gates”. This awareness will affect society in general and manifest later when these students are employed. “The key is to develop students who do not want to cheat” (Larkin et al. 2017, p.6). Not only that, but we might need to re-define Academic Integrity for the digital age and amend policy accordingly (Reedy et al. 2021).

Second, faculty and administration should eliminate and reduce opportunities for students to cheat through employing certain exam features as suggested by Olt (2002) and Reedy et al. (2021), where it is made difficult for them to cheat in online exams. This should be in addition to designing meaningful instruments and assignments and using robust techniques to assess students’ knowledge rather than information recall, when appropriate.

Finally, faculty and those responsible for administering exams should be given extensive training on cheating methods and techniques employed by students as well as on what ignoring the practice may lead to. Furthermore, this training “must be renewed and adapted to the current and real needs of university teachers and university education” (Alvarez et al. 2009, p.333). Downplaying the problem is not in the interest of any stakeholders, especially because Higher Education (HE) is where student identity is created and formed. Hence, this issue should be dealt with effectively. Furthermore, policymakers and those in higher administration should not shy away from applying severe punishments for cheating and academic dishonesty to send a clear message of the gravity of this practice and deter those who might be presented with the opportunity to engage in cheating and plagiarism from doing so online or otherwise (Chace 2012, p.30):

Assuming that something should be done, one response could be to stiffen the apparatus of policing. Internet sites such as "Turnitin," to which students and teachers can submit student work to see if it contains material from essays already on electronic file, could be employed by more and more teachers to track down those who misuse the material. Penalties could be increased;

Only when all these factors are taken into account can a secure and valid assessment take place.

### Limitations of the study

Despite the study providing some insight about some of the challenges of administering tests online and factors leading to ease in cheating and plagiarizing when tests primarily involve recall, our study had a number of limitations. Hence, when discussing the results, a few main points must be considered carefully. This research described a special situation that was very difficult to forecast, unlike studies designed to assess e-learning systems’ efficiency before the pandemic. These questionnaires were created during the time in which the lockdown was taking place, several things were unknown, educational and social changes were occurring on a regular basis, and there were fears that once the summer break began, we would not be able to reach out to students and faculty. Most of the questions and design were a direct response to what was taking place during the lockdown period of the COVID-19 emergency, and we began collecting answers at once, although more time to reflect on the tools would have improved the robustness of the instruments during the data collection phase. Moreover, this study employed descriptive statistics, which only provided synopses about the individuals or variables under study; therefore, we need to be cautious when generalizing the results generated from the questionnaires to other individuals or universities. Furthermore, upon reflection, it would have been much better to have included a sub-set of targeted qualitative questions instead of qualitative data based only on the responded to the ‘other’ options given in some survey questions.

One strength of this study is that it enriches previous studies with students’ and teachers’ perspectives on assessment practices online during an emergency period; however, this study provided insights into cheating methods and reasons through student self-reporting that can be difficult to verify due to this topic’s sensitive nature despite assured anonymity. At faculty level, we have to stress teachers’ perceptions of the need for assessment literacy training, and at policy level, we have to emphasize the application of policy regarding cheating and plagiarism through the development of a clear code of ethics and the dissemination of that information throughout the university. Overall, the results of this study offered new insights into the adoption of e-learning and online assessment during an emergency situation, such as the COVID-19 pandemic of 2020.

### Future research

This study aimed to bring a deeper understanding of student and faculty perspectives of online assessment during COVID-19 and can be a sound basis for future research. For example, future research should involve more universities and more teachers in different contexts from around the world. Secondly, using the faculty questionnaire (with minor changes) with both students and faculty might yield a more comparable view. Thirdly, the current study focused on student and faculty perspectives; future research could focus on the institutional level and methods for raising student moral and ethical standards is a matter worthy of future research as well. Comparison with similar surveys done later in the pandemic, when the shift did not feel quite so much as an emergency and students got settled into the ‘new normal’, could prove interesting.

## Data Availability

Student data are in Arabic, while faculty data are in English and both are available upon request.

## Abbreviations

KAU: King Abdulaziz University
MoE: Ministry of Education
LMS: Learning Management System
PYP: Preparatory Year Program
WHO: World Health Organization
